# Child maltreatment and ADHD symptoms in a sample of young adults

**DOI:** 10.3402/ejpt.v7.32061

**Published:** 2016-06-14

**Authors:** Karoline Sanderud, Siobhan Murphy, Ask Elklit

**Affiliations:** Department of Psychology, National Center of Psychotraumatology, University of Southern Denmark, Odense, Denmark

**Keywords:** Abuse typologies, child maltreatment, attention deficit hyperactivity disorder

## Abstract

**Objective:**

The present study investigated the relationship between different types of childhood maltreatment (emotional, sexual, overall abuse, and no abuse) and the symptoms of attention deficit hyperactivity disorder (ADHD) in young adulthood.

**Method:**

Data were collected from a Danish national study conducted by The Danish National Centre for Social Research in 2008 and 2009. A sample of 4,718 young adults (24 years of age) were randomly selected using the total birth cohort of children born in 1984. Structured interviews were conducted with a response rate of 63%, equating to a total sample size of 2,980 participants.

**Results:**

Chi-square analyses revealed significant relationships between child maltreatment groups and a probable diagnosis of ADHD using the Adult ADHD Self-Report Scale (ASRS). Binary logistic regression analysis showed that the overall abuse class was more strongly associated with probable ADHD (OR=5.08), followed by emotional abuse (OR=3.09) and sexual abuse (OR=2.07).

**Conclusions:**

The results showed that childhood maltreatment was associated with increased risk of ADHD symptoms in young adulthood. The findings of this study are discussed within the existing literature and suggestions for future research are outlined in order to replicate these findings in other adult populations.

**Highlights of the article:**

- Child maltreatment is associated with higher levels of ADHD symptoms in a nationally representative sample of young Danish adults.
- Co-occurring (multiple) types of maltreatment display stronger associations with ADHD symptoms with attenuated effects for sexual abuse.
- Males confer stronger associations with ADHD symptoms than females.
- Findings underscore the importance of exploring the role of childhood trauma in young adults with ADHD symptoms.

The effects of abuse on a child's development and behavior are complex and multifaceted. Several factors can potentially harm and disrupt the natural development of children, and child maltreatment covers a broad spectrum of factors that can affect a child's emotional and psychological wellbeing. According to developmental theory and research (Perry, 2006), child maltreatment affects sensory, cognitive, and affective modalities. The developmental and cognitive issues typically seen in maltreated children have similarities to the problems reported of attention deficit hyperactivity disorder (ADHD) youth. These similar features of ADHD and maltreated children include aggression and externalizing behavior, depression, and cognitive difficulties (Briscoe-Smith & Hinshaw, 2006), which suggests there is a possible association between child maltreatment and ADHD. This study focuses on several forms of child maltreatment, and the association with ADHD symptoms in young adults.

Many individuals, who were previously defined as having a combination of attentional and behavioral problems, are now receiving a clinical diagnosis as ADHD, and being treated with prescription drugs. The prevalence of ADHD in Denmark among children 0–5 years old has increased by more than 600% in the last 10 years (Langager, 2014). Additionally, more than 35,500 people in Denmark are registered for prescription medication to treat ADHD. ADHD is characterized by high emotional disturbance, self-regulatory problems, and poor attention skills (American Psychiatric Association, 2010), and the increasing numbers of children diagnosed with the disorder has attracted a lot of research and media attention. The classical symptoms of ADHD lie on a spectrum ranging from normal to very disruptive, and it is often the external symptoms such as high impulsivity and irritability that initiate medical attention. Diagnosing ADHD, however, is not a clear-cut process due to the high comorbidity with mood and anxiety disorders (Weinstein, Staffelbach, & Biaggio, 2000).

Several studies have revealed significant associations between ADHD and some types of childhood maltreatment. Additionally, ADHD symptoms have been compared with posttraumatic stress disorder (PTSD) in order to find distinguishing and overlapping symptoms (Ford et al., 2000). Victims of abuse and individuals with ADHD share common features of behavioral problems, peer rejection, and cognitive difficulties (Briscoe-Smith & Hinshaw, 2006), therefore suggesting there may be an association between the two disorders. Cuffe, McCullough, and Pumariega (1994) highlighted the comorbidity of the two disorders using case studies of traumatized children. Differentiating the two disorders, in addition to examining their diagnostic overlap, has important treatment implications. Besides, one might also explore the possible etiologic and/or exacerbating role of childhood maltreatment in the development of ADHD symptoms.

Ouyang, Fang, Mercy, Perou, and Grosse (2008) investigated the association between different types of ADHD and different types of child maltreatment, and concluded that compared with the non-ADHD category, individuals with any form of ADHD were significantly associated with four types of child maltreatment (supervision neglect, physical neglect, physical abuse, and contact sexual abuse). This suggests that ADHD is more likely among children with a history of abuse; however, it is difficult to establish whether an ADHD diagnosis and symptoms were present before or if it was a result of the abuse.

Briscoe-Smith and Hinshaw (2006) found significantly higher rates of abuse for girls with ADHD (14.3%) compared with their peers (4.5%). Likewise, Ford et al. (2000) found that ADHD increased the likelihood of exposure to physical or sexual maltreatment; 25% of the children with ADHD diagnosis had been exposed to physical maltreatment and one in nine had been exposed to sexual maltreatment. This study examined the relationship between both ADHD and oppositional defiant disorder (ODD) and childhood abuse. The results found that 91% of individuals with comorbid ADHD and ODD had a history of trauma compared to the ADHD only group. This indicates a high prevalence level of maltreatment among the comorbid ADHD and disruptive behavior group compared to the control group of typically developed children (Ford et al., 2000). Using a population-based sample, Fuller-Thomson, Mehta, and Valeo (2014) found that those who reported abuse were seven times more likely to have ADHD than those who were not abused. The authors also highlighted the similarity in risk factors (e.g., parental unemployment, addiction, and divorce) between physical abuse and ADHD.

There are several limitations of previous studies examining the association between childhood abuse and ADHD that this study intends to build upon. First, most research has focused on one type of childhood abuse and not considered the potential of multiple forms of co-occurring abuse. Second, the studies discussed above have used various methods to determine ADHD symptoms; however, the diagnosis is difficult to assess, and classification of ADHD can vary from country to country or even from clinician to clinician. Likewise, the definition of abuse is significant when determining whether there is a relation to ADHD or not, and which form of abuse has the strongest relationship. Third, there is also a paucity of research that focuses on ADHD in young adults with the majority of research attention dedicated to childhood samples. Finally, few studies have used a nationally representative sample when researching the relationship between ADHD and abuse.

## Aims of the present study

The current study therefore aimed to examine the association between typologies of child maltreatment established from latent class analysis (Armour, Elklit, & Christoffersen, 2014), as well as co-occurring maltreatment and ADHD symptoms in adult life. Providing a representative sample, this study could present more robust evidence for the results. Using specific questions regarding experiences in early life and self-reported ADHD symptoms, this study also aims to examine what type of child maltreatment is associated with greater risk of adult ADHD symptoms. There are several notable analytical and conceptual benefits of utilizing typologies of child abuse based on latent class analysis by offering more precise defined clusters of abuse than used previously. Therefore, stronger estimates of the association between ADHD symptoms and childhood maltreatment can be made.

## Methods

### Procedure

A stratified random probability survey was conducted in Denmark between 2008 and 2009. Statistics in Denmark randomly selected 4,718 participants born in 1984. The Danish Research Council founded the study that was approved by the Danish Data Protection Agency. To increase the number of participants who had experienced childhood abuse and neglect, children who had been assigned child protection service status by the Danish authorities was oversampled using a 1:2 ratio (*n*=850). A child protection case was objectively defined as a case where the council had provided support for the child and the family or placed the child with a foster family due to concerns about the wellbeing and development of the child. This assessment took place according to files of the local social workers.

Data were collected using a structured interview on entirely voluntary participants, conducted either over the phone or at home. Trained interviewers conducted the interviews with an average duration of 43 min. An option to fill out sensitive questions alone on a laptop was given to the interviewee during the interview, while the interviewer waited in a different room. Post-interview psychological help was offered to all respondents via a telephone helpline. The second author served the helpline.

### Participants

A total of 2,980 interviews were successfully conducted, equating a response rate of 63%. 850 interviews were conducted with individuals who had been previously identified by the Danish authorities as child protection services cases; the survey data and analyses were weighted to the account of this oversampling. Individuals who had previously refused to participate in national research, who were either incarcerated, thought to have severe learning difficulties, or had moved out of the country were excluded. The most common reasons for non-participation were refusal to take part in the study (21%), being unreachable (13%), or illness or disability (2%). The sample consisted of 1,579 males (52%) and 1,401 females. The majority of the sample either owned or rented their own private accommodation (93.7%), and almost half was married or cohabiting (46.0%).

### Measures

Participants were asked 20 questions across four domains of childhood maltreatment: physical abuse (e.g., “Have you ever been beaten with an object, such as a whip or coat hanger”); psychological abuse (e.g., Have you ever been addressed in a humiliating [being called lazy, stupid, or useless] manner by parents/guardians); neglect (e.g., Were you ever occasionally starved due to lack of food or no one available to prepare meals); and sexual abuse (e.g., Did you ever experience forced/completed intercourse). Four latent classes of abuse emerged: non-abused, emotionally abuse, sexual abuse, and abused overall. Retrospective reports on abuse or neglect by the parents or guardians were obtained on all four of these latent classes, utilizing items that described different experiences of physical neglect, emotional abuse, neglect, or serious sexual abuse (Armour et al., 2014).

ADHD symptomatology was measured through six short screening items (see Table 1) reflecting some core symptoms of ADHD: concentration problems, hyperactivity, and memory problems based on the Adult ADHD Self-Report Scale (ASRS v1.1; Kessler et al., 2005). This measure is based on the criteria for ADHD from the DSM-IV-TR (American Psychiatric Association, 2010) and measures the frequency of ADHD symptoms. The response format is rated on a 5-point Likert scale of 1=“never,” to 5=“all the time.” The ASRS items were recoded such that a symptom was rated as present if the item corresponding to the symptoms was scored ≥3 in the first three questions and ≥4 in the remaining three questions. To establish overall probable ADHD a binary variable was created (ranging from 0 to 6) using a threshold of ≥4 to reflect probable adult ADHD (Schweitzer, Cummins, & Kant, 2001).

**Table 1:** ASRS; items for ADHD in adults

How often do you experience difficulties completing a project and get the last details in place when the hardest task of the work is already done? How often do you experience difficulties finishing a task that requires planning? How often do you have problems remembering appointments and such that you should remember? How often do you avoid of put off a task that requires several considerations? How often do you experience problems sitting still with your hands and feet, when you are supposed to sit down for a longer period of time?
How often do you feel overactive and in need of moving, like you were driven by a motor?

The ASRS has underwent psychometric testing and has been validated as a reliable screening measure in adolescent and adult samples (Adler et al., 2006, 2012; Reuter, Kirsch, & Hennig, 2005). The reliability estimate in the current sample was acceptable (Cronbach alpha α=0.66).

## Results

A one-way analysis of variance (ANOVA) was conducted on the abuse typologies and overall ADHD score. The results indicated significant differences between the abuse groups on ADHD scores *F* (2, 2,939)=40.07, *p*<0.001). The overall abuse group reported higher levels of ADHD symptoms (M=16.87, SD=4.30) with minimal differences to the emotional abuse group (M=16.01, SD=3.93) and sexual abuse group (M=15.30, SD=3.95). Post hoc analysis indicated that only the non-abused group differed significantly from the three abuse groups with much lower levels of ADHD (M=13.81, SD=3.72).

A total of 431 (14.5%) participants were above the cut off, scoring symptoms compatible with a probable ADHD diagnosis. A Chi-square analysis indicated significant association between childhood trauma groups and ADHD symptoms [χ^2^(3) =88.44, *p*<0.001]. Cross tabulations revealed that the overall abuse latent class was especially associated with ADHD symptoms, with a total of 35.9% scoring higher than the cut off score on the ASRS (see Table 2). Furthermore, all three abuse categories had a higher total group percentage within the cut off for ADHD in adults (emotional abuse: 25.5%, sexual abuse: 15.3%) than the non-abused group (10.1%). This suggests a history of abuse confers a higher risk of developing ADHD symptoms in later life compared to non-abused individuals as evidenced by the overall abused group containing more than three times as many individuals with ADHD symptoms than the average population.

**Table 2:** ADHD and childhood abuse categories

	Emotionally abused	Sexually abused	Abused overall	Non-abused	Total
No ADHD	196	50	41	2,329	2,616
% without ADHD	74.5%	84.7%	64.1%	89.0%	87.9%
ADHD	67	9	23	262	361
% with ADHD	25.5%	15.3%	35.9%	10.1%	12.1%
Total	263	59	64	2,591	2,977

Table 3 reveals the results of the binary logistic regression analysis with ADHD as the dependent variable, gender, and the three abuse classes as independent variables and using the non-abuse class as the reference category. The overall model was statistically significant [χ^2^ (4)=82.81, *p*<0.001]. Males were significantly more likely than females to report probable ADHD (OR=1.56). The three abuse classes showed a significantly increased risk of developing ADHD symptoms relative to the non-abused class. The strongest effect was evident in the overall abuse class with over a fivefold increase in probable ADHD. Participants in the emotional abuse class were over three times more likely to develop ADHD symptoms and in the sexually abused class were twice as likely to report probable ADHD.

**Table 3:** Odds ratio for gender and latent classes, in developing ADHD symptoms

			Confidence interval	
			
	B (SE)	Odds ratio	CI lower	CI upper
Gender (male)	0.44 (0.12)***	1.56	1.23	1.96
Emotional	1.13 (0.16)***	3.09	2.27	4.20
Sexual	0.73 (0.37)*	2.07	1.00	4.29
Overall	1.63 (0.27)***	5.08	2.98	8.66

*** *p*<0.001

* *p*<0.05.

B=beta, SE=standard error.

## Discussion

The aim of this study was to explore the association between different types of childhood maltreatment and ADHD symptoms. Similar to previous research, the results revealed a strong relationship between survivors of different kinds of childhood abuse and ADHD indicators. Overall, individuals whom had self-reported history of abuse also reported higher ADHD symptoms.

The findings indicate that different forms of child maltreatment conferred a 2- to 5-fold increase in risk for adult ADHD symptoms. A possible explanation for these findings is that the potential symptom domains (attention, impulse control, and hyperactivity) are comparable in both ADHD and PTSD (Ford & Connor, 2009). This underscores that it may be difficult to decipher whether symptoms are the result of PTSD or ADHD. However, the core ADHD symptoms such as “problems sitting still” and feeling “overactive and in need of moving” were endorsed frequently (≥4) by 36 and 21% in the current sample. Although some researchers have identified commonalities in symptom expression between ADHD and PTSD and highlight the potential of misdiagnosis (Ford et al., 2000), these results signify the importance of fully elucidating the problems individuals with history of abuse experience. Based on the ASRS and the proven validity of its results, the current results suggest that ADHD warrants more research attention in child maltreatment literature. Examining whether ADHD may be a problem after experiencing childhood maltreatment would help inform targeted treatment for this group. In addition to environmental factors, ADHD has strong genetic factors and influences of etiology (Asherson, 2013). Externalized behavior problems typical for individuals with ADHD may reflect a trauma history and it is to be anticipated that youth with such history may lack internal coping strategies that typically develop throughout childhood.

The relationship between abuse and ADHD has previously been discussed in literature (Briscoe-Smith & Hinshaw, 2006; Cuffe et al., 1994; Ford et al., 2000; Fuller-Thomson et al., 2014; Ouyang et al., 2008). However, the current results reveal variations depending on type of abuse. It is interesting that individuals in the abused overall group reported the highest number of ADHD symptoms, which could suggest individuals in this class have a higher probability of developing ADHD in later life. This may be due to the severe nature and number of types of abuse in this group, which may induce negative outcomes on several domains of the child's life (Finkelhor, Ormrod, & Turner, 2007, 2009). Another possibility is individuals in this group displayed higher levels of physical abuse in comparison to the other maltreatment groups, which highlights the potential role of physical abuse and ADHD symptoms that has been evidenced in previous studies (Ford et al., 2000). This finding further indicates that more research is needed to delineate the specific effects of sexual and physical abuse independently on symptoms of ADHD due to the high levels of co-occurrence of both types of maltreatment.

Additionally, the individuals in the emotional abuse group reported second strongest association to ADHD. Possible mechanisms involved are the high emotional disturbance ADHD youth experience in several domains of life, which may be a result of emotional abuse in childhood. Emotional abuse has a direct effect on social support, self-esteem, and increased risk of being bullied at school (Murphy, Shevlin, Armour, Elklit, & Christoffersen, 2014). All of these variables can be involved in the disruptive behaviors seen in ADHD youth. There is a paucity of research on emotional abuse which may be the result of difficulties in detection. Further, this type of abuse does not meet the criteria as a traumatic stressor, which may be another reason why this type of abuse often neglected. However, the profound consequences of emotional abuse have recently been identified (Elklit, Karstoft, Feddern, & Christoffersen, 2013; Roenholt, Beck, Karsberg, & Elklit, 2012). The current findings therefore support these studies by demonstrating the higher levels of ADHD symptoms in children exposed to emotional abuse in comparison to those who experienced sexual abuse and non-abused controls. Notably, another possible explanation for these findings is that individuals with ADHD symptoms may recall childhood relationships as emotionally abusive. Similarly, the behavioral patterns of children with ADHD symptoms may elicit emotionally abusive responses from caregivers.

Overall, all of the maltreatment groups reported higher levels of ADHD symptoms. Future research could examine specific symptoms to further delineate how maltreated children report higher levels of ADHD symptoms. Mechanisms such as hypervigilance and restlessness may be a way of coping with the abuse, as well as related to the hyperactivity seen in ADHD youth. Further, as not all ADHD youth display hyperactivity, future research should look into the subgroups of ADHD related to different forms of maltreatment, as well as internal and external behaviors.

As symptom expression and the development of symptoms vary in individuals, education and health care professionals may benefit from assessing childhood abuse in students experiencing ADHD-type problems. With ADHD symptoms being such a high potential risk factor for individuals with child abuse histories, focus should lie on the diagnostic importance of the history of the child, as well as the underlying factors behind the symptom expression. In the same way, as educators and practitioners working with ADHD youth could benefit from having a trauma informed assessment, those working with maltreated children should be informed about the high association between abuse and ADHD symptomatology. For children with histories of abuse, therefore, it may be helpful to screen for ADHD in order to improve the treatment process.

Treatment will naturally be different if the ADHD symptoms are a consequence of repressed memories from abuse. Symptoms such as inattentiveness and difficulty concentrating are associated with all types of abuse (Ouyang et al., 2008), and have a high similarity to ADHD. Additionally, the societal cost of ADHD children is high, and if the treatment or the measures taken for this group are not beneficial because there has never been an extensive background check involving child abuse, the cost for society will only increase. Attention to the association between ADHD and abuse should therefore be a priority in order to decrease wrongful treatment or diagnoses and to lower the costs for society.

Further research should focus on whether ADHD is a consequence or a potential risk factor of childhood abuse. Additional research is also necessary regarding the treatment process for those individuals with abuse histories and ADHD symptoms. Because of the now known association, future research should determine the subgroups of ADHD in relation to abuse classes and the different symptom expression. This will in turn be helpful in diagnostic purposes and for individuals working with (groups of) either ADHD youth or abused children.

### Limitations

This study is not without limitation. First, several definitions of maltreatment and abuse have been presented in literature, presenting problems comparing the present study with previous findings in the child maltreatment field. The definitions of ADHD are even more diverse, and the diagnosis is used widely and across several dimensions of severity. This study also cannot determine specifically if the individual's responses lead to an ADHD diagnosis, as many of the examined symptoms can relate to a range of psychiatric disorders, for example, anxiety disorders, PTSD, or ODD (Ford et al., 1999, 2000; Weinstein et al., 2000). Further, this study is based solely on self-report answers which may lead to individuals over- or underreporting their symptoms. The study is also subject to recall bias; however, with this national representative sample, there is reason to believe that the validity is strong and the findings can be generalized.

## Conclusions

The present study utilized a latent class analysis to identify a meaningful set of latent subgroups characterized by different experiences of childhood maltreatment. This provided an opportunity to examine outcomes that may be associated with certain types of maltreatment Additionally, co-occurrence of two or more types of abuse often happens, hence resulting in a more severe and broad range of possible consequences. Finally, this study presented the opportunity to examine the association between childhood maltreatment and symptoms of ADHD in young adults using a large national representative sample.

## Funding statement

The work was no supported by any funding agency.
